# The microbiome of common bedding materials before and after use on commercial dairy farms

**DOI:** 10.1186/s42523-022-00171-2

**Published:** 2022-03-07

**Authors:** Tui Ray, Tara Nath Gaire, Christopher J. Dean, Sam Rowe, Sandra M. Godden, Noelle R. Noyes

**Affiliations:** 1 Department of Veterinary Population Medicine, University of Minnesota, St. Paul, 55108 USA; 2 Sydney School of Veterinary Science, The University of Sydney, Camden, NSW 2570 Australia

**Keywords:** Bedding, Differential abundance, Mastitis pathogens, Microbiome

## Abstract

**Background:**

Bovine mastitis is one of the most economically important diseases affecting dairy cows. The choice of bedding material has been identified as an important risk factor contributing to the development of mastitis. However, few reports examine both the culturable and nonculturable microbial composition of commonly used bedding materials, i.e., the microbiome. Given the prevalence of nonculturable microbes in most environments, this information could be an important step to understanding whether and how the bedding microbiome acts as a risk factor for mastitis. Therefore, our objective was to characterize the microbiome composition and diversity of bedding material microbiomes, before and after use.

**Methods:**

We collected 88 bedding samples from 44 dairy farms in the U.S. Unused (from storage pile) and used (out of stalls) bedding materials were collected from four bedding types: new sand (NSA), recycled manure solids (RMS), organic non-manure (ON) and recycled sand (RSA). Samples were analyzed using 16S rRNA sequencing of the V3–V4 region.

**Results:**

The overall composition as well as the counts of several microbial taxa differed between bedding types, with *Proteobacteria*, *Actinobacteria*, *Bacteroidetes* and *Firmicutes* dominating across all types. Used bedding contained a significantly different microbial composition than unused bedding, but the magnitude of this difference varied by bedding type, with RMS bedding exhibiting the smallest difference. In addition, positive correlations were observed between 16S rRNA sequence counts of potential mastitis pathogens (bacterial genera) and corresponding bedding bacterial culture data.

**Conclusion:**

Our results strengthen the role of bedding as a potential source of mastitis pathogens. The consistent shift in the microbiome of all bedding types that occurred during use by dairy cows deserves further investigation to understand whether this shift promotes pathogen colonization and/or persistence, or whether it can differentially impact udder health outcomes. Future studies of bedding and udder health may be strengthened by including a microbiome component to the study design.

**Supplementary Information:**

The online version contains supplementary material available at 10.1186/s42523-022-00171-2.

## Background

Bedding management has been a crucial component of dairy farming. Ideally, dairy cows should spend about 10 to 13 h per day in a prone position to encourage essential physiological activities such as rest and rumination [1, 2]. The bedding material on which dairy cows rest has been shown to impact cow comfort and productivity, and proper bedding management plays an important role in increasing the productivity of dairy farms [3]. Choice of bedding material is one crucial aspect of bedding management, and the type of bedding has been shown to have a significant effect on udder health and production outcomes in dairy cows [4].

Bedding materials can be broadly classified into two main groups: inorganic and organic, with the latter category subclassified into non-manure organic materials and manure-based materials [5]. Recent studies reported that inorganic materials were the most common bedding type used by U.S. dairy farms, followed by organic non-manure materials, and finally manure-based materials [6]. However, these studies comprised convenience samples, and the true distribution of bedding material use on U.S. dairy farms is not currently known, particularly by herd size. Organic bedding materials are typically composed of plant byproducts such as straw, hay, saw dust, wood shavings, crop residues, and composted manure or dried manure solids [7]. Availability and low cost make these materials a popular bedding choice, while a major drawback is that they promote rapid growth of environmental mastitis pathogens after getting mixed with fresh manure and moisture in dairy farms [8]. In contrast to organic bedding, inorganic bedding materials are not made from plants or other organic materials. Sand is the most common inorganic bedding type and is considered to be the gold standard of bedding materials because new (virgin) sand is relatively dry and should contain very low levels of organic matter. As such, bacterial growth is impeded, and mastitis causing pathogens are often significantly lower in used sand bedding compared to organic bedding material [9]. Sand also provides superior comfort [10]. However, sand can be more costly than some other bedding materials, depending on local availability. Recycling and reusing sand bedding can help to reduce this cost, but does not alleviate other complications from sand, including disadvantages during manure handling when the sand settles at the bottom of manure collection pits.

Bedding management practices can greatly affect the cleanliness and bacterial population of bedding on dairy farms. The amount and application frequency of fresh bedding are two management factors that impact the bedding microbiome, i.e., the microbial population on the bedding. Organic bedding materials usually reach maximum bacterial populations within 24 h after the new material is laid down [11, 12]. Moisture and pH also influence bacterial growth in bedding materials [8], and infrequent bedding replacement allows for more accumulation of manure, mud and urine which can rapidly deteriorate bedding quality, leading to extensive contamination.

Bacterial growth also varies between different bedding types depending upon the physical, biochemical, and nutritional characteristics of the bedding [9]. Previous studies found that a higher percent of bedding dry matter was associated with reduced total bedding bacterial counts; and that frequent addition of new bedding material into used bedding improved cow hygiene [13]. To evaluate bedding quality and its relation to mastitis in dairy cows, multiple studies have evaluated the total bacterial count and presence of common pathogens in various bedding materials. While certain mastitis pathogens can be considered innate to some types of bedding, others, such as *E. coli* or *Klebsiella* spp., are assumed to be introduced through contamination of bedding materials by feces, water, or feed [14]. Different types of bedding have exhibited different levels of both total bacterial counts and counts of bacteria such as *Bacillus* spp., *Klebsiella* spp., coliforms and non-coliform gram-negative organisms, streptococci or *Streptococcus*-like organisms (SSLO), and *Staphylococcus* spp. [6, 15]. While most studies have focused on mastitis-causing pathogens and total bacterial counts derived from aerobic culture, few reports describe a predominance of other pathogens belonging to the families *Aerococcaceae*, *Ruminococcaceae*, *Moraxellaceae*, *Corynebacteriaceae*, *Staphylococcaceae* and *Lachnospiraceae* [16, 17].

Intramammary infection (IMI) is a prevalent problem in dairy production, causing huge economic loss for dairy producers and negatively impacting cow health and milk quality. Bedding materials have been associated with mastitis epidemiology [18, 19]. Numerous studies have demonstrated a correlation between bedding bacterial counts (BBC) and the counts of bacteria on the teat apex of cows using that bedding, suggesting that bedding may be a substantial source of bacteria colonizing the teat epithelium [8, 20–23]. Molecular epidemiologic studies have identified IMI-causing strains of bacteria in bedding material, suggesting that bedding can act as a reservoir for some pathogens [24, 25]. Aerobic culture of bedding to determine BBC has been used to estimate bedding-associated mastitis risk [5, 6]. Furthermore, it has been suggested that certain types of bedding materials have been shown to increase mastitis risk due to propensity to support pathogen growth, which then colonizes the teat, leading to infection [6]. Though many studies have demonstrated a correlation between BBC and teat end bacteria count, and between BBC and mastitis risk [6, 26], few studies have investigated potential association between the bedding microbiome and mastitis. Furthermore, it is unknown whether the commensal bedding microbiome plays a role in supporting or preventing colonization of the bedding with potential mastitis pathogens. There are descriptive reports of various aspects of the bedding microbiome, including seasonal variation [16] and changes associated with manure solids recycling [27]; however, none compare the culturable and unculturable microbiome of different types of bedding in relation to use status.

Very little is known about the bedding microbiome, including whether or not it differs by bedding type and during use by cows. Advancing baseline knowledge of the bedding microbiome is a first step towards understanding whether and how the bedding microbiome either supports or degrades udder health and pathogen control. Therefore our objectives were to (a) describe and compare microbial community structure (including potential mastitis pathogens) across common types of bedding materials from the U.S. dairy farms, utilizing culture-independent 16S rRNA sequencing; (b) determine whether use of the bedding by dairy cows alters the bedding microbiome and/or potential mastitis pathogens as measured using 16S rRNA sequencing; and (c) evaluate whether 16S rRNA counts of potential mastitis pathogens correlate with aerobic culture-based total and pathogen-specific bacterial counts.

## Results

### Results of 16S rRNA sequencing of bedding samples

Complete metadata for each analyzed sample can be found in Additional file 1: Table S1. Sequencing of the V3–V4 hypervariable region of the 16S rRNA gene on the Illumina MiSeq platform generated a total of 7.4 M paired-end sequence reads across all 88 samples, including negative and positive controls (mean 82 K per sample, range 1.3–123 K). The negative and positive control samples yielded 2.5 K and 3.9 K raw reads, respectively. Average number of raw sequences generated for RMS, NSA, ON, and RSA samples were 88 K, 71 K, 84 K, and 84 K, respectively, and these differences were not statistically significant based on regression modeling (ANOVA *P* = 0.40). However, used bedding samples yielded significantly more raw reads on average than unused bedding samples (β_used_ = 11,554 reads, 95% CI =  − 435 to 22,672 reads, ANOVA *P* = 0.04). After quality filtering, 5.1 M sequences remained across all samples; and after merging the forward and reverse sequence reads and removing chimeras, 4.7 M paired-end sequences remained (Additional file 1: Table S1). Six bedding samples produced very low numbers of reads (Additional file 1: Table S1), which was expected given that these six samples also yielded low total DNA and low 16S rRNA gene copy number as determined by qPCR. All six of these samples originated from unused NSA, ON and RSA beddings, which may have accounted for the very low microbial biomass. Two of these low-biomass samples contained fewer reads than the negative controls and were therefore removed from further analysis. After removing controls and the two outlier samples, the distribution of per-sample reads (after quality control and filtering) ranged from 20 to 90 K for most samples, with a mean of 54 K reads per sample. Furthermore, the number of raw reads per sample was no longer significantly different by bedding type or bedding status (ANOVA *P* = 0.74 and 0.14, respectively), confirming that the two very low-yielding unused bedding samples had been significantly influencing the distribution of reads across used and unused samples. After removal of these samples, sequencing effort was evenly distributed across bedding types and status, and therefore sequencing depth was unlikely to introduce systematic bias into the analysis.

Across all sequenced samples, a total number of 31,576 ASVs were identified. Among these, 198 were identified as potential contaminants by decontam. As expected, these ASVs represented a very small number of sequence counts, i.e., 27,343 out of 4.6 M sequences. Following removal of these sequence features, 31,378 ASVs from 86 samples remained for downstream analysis. Analysis of the positive control spike-in sample against the complete SILVA database showed *Truepera* as the most abundant genus with 51% of all reads, and *Imtechella* as the third-most abundant with 8.5% of reads. The genus *Allobacillus* was not identified, but the SILVA database only contains one reference for *Allobacillus halotolerans*. Therefore, we also aligned the sequences from the mock community to a custom database provided by ZymoBIOMICS (see “Methods” section), which resulted in detection of all three expected taxa, with *Truepera radiovictrix* comprising 33.8% of reads, *Imtichella halotolerans* 60.3%, and *Allobacillus halotolerans* 5.9%.

### Bacterial community composition across bedding types and status

**Fig. 1:**
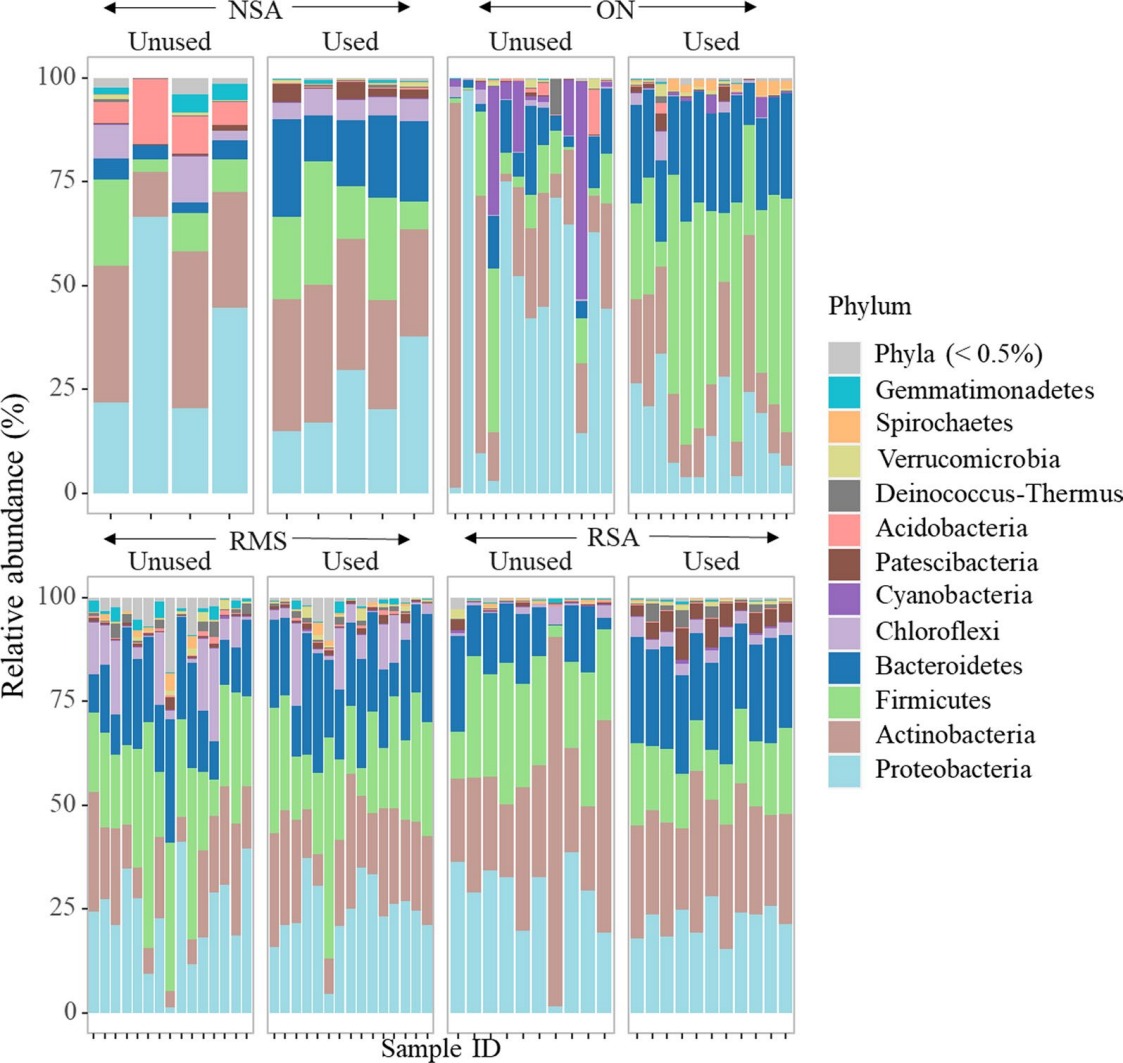
100% stacked relative abundance bar plots for each bedding sample, at the phylum level, grouped by bedding status and type. Phyla comprising < 0.5% of the total sequence counts were grouped together. *NSA* new sand, *ON* organic non-manure, *RMS* recycled manure solids, *RSA* recycled sand

Of the 31,576 ASVs identified, 31,532 ASVs were classified as bacteria; 27 as eukaryota; 1 as archaea; and 16 remained uncharacterized at the kingdom level. As expected, the percentage of classified ASVs decreased stepwise with increased taxonomic resolution, from 98.8% at the phylum level down to 3.5% at the species level (Additional file 1: Table S2). Given the low classification rate at the species level, we performed all subsequent analyses at the genus level and higher. Detailed species-level results are available in Additional file 1: Table S3. Taxonomic evaluation of the bedding microbiome across all samples revealed that *Proteobacteria*, *Firmicutes*, *Actinobacteria*, *Bacteroidetes*, *Chloroflexi*, *Cyanobacteria* and *Patescibacteria* were the most abundant phyla, accounting for 95.6% of the total sequence reads, with differential abundance by bedding type and status (Additional file 1: Table S4). For visualization purposes, we grouped together low abundance phyla (i.e., those comprising < 0.5% of the sequence counts at the phylum level), and then compared the phylum-level profile between used and unused bedding materials (Fig. 1). In both unused and used NSA, *Actinobacteria*, *Proteobacteria* and *Firmicutes* were the dominant phyla, accounting for more than 70% of the sequence counts. *Acidobacteria* was also a dominant phylum in unused NSA, but it was largely absent in used NSA bedding samples (Fig. 1). Conversely, *Bacteroidetes* comprised a larger proportion of the phylum-level microbiome in used versus unused NSA. Used ON bedding exhibited a considerable increase in Firmicutes compared to the unused ON (Fig. 1), whereas *Proteobacteria* exhibited a relative decrease in used versus unused ON bedding. Both unused and used RMS bedding showed predominance of *Proteobacteria*, *Firmicutes*, *Bacteroidetes* and *Actinobacteria*, contributing to more than 80% of the phylum-level sequence counts. Unlike with ON and NSA, the phylum-level profile of the RMS bedding samples did not shift dramatically between used and unused status. As with RMS samples, RSA bedding was dominated by the same four phyla and also exhibited little difference in abundance between unused and used status.

The most abundant class-level taxa across all samples were *Actinobacteria*, *Gammaproteobacteria*, *Bacteroidia*, *Bacilli*, *Clostridia*, *Alphaproteobacteria* and *Chloroflexia*, which together comprised 86% of the total reads across all samples. The most abundant order-level taxa were *Micrococcales*, *Pseudomonadales*, *Clostridiales*, *Bacteroidales*, *Flavobacteriales*, *Bacillales*, *Lactobacillales* and *Corynebacteriales*. At the family level, the most abundant taxa were *Pseudomonadaceae*, *Moraxellaceae*, *Flavobacteriaceae*, *Corynebacteriaceae*, *Intrasporangiaceae*, *Ruminococcaceae*, *Micrococcaceae*, *Sphingobacteriaceae* and *Aerococcaceae*, while approximately 7% of the reads remained uncharacterized at the family level. Forty-five family-level taxa had a relative abundance greater than 0.5%, accounting for more than 73% of the reads. The most abundant genera were *Pseudomonas* (gram-negative, 4.8% of all sequence reads), *Corynebacterium_1* (gram-positive coryneform, 3.7%), *Acinetobacter* (2.4%), *Psychrobacter* (gram-negative cocci, 2.3%), and *Ornithinimicrobium* (gram-positive rod shaped, 1.7%).

The four non-outlier yet low-yielding bedding samples were dominated by varying bacterial phyla (Additional file 1: Table S5). The unused NSA outlier sample was dominated by *Proteobacteria* (66% of all reads), followed by *Acidobacteria*, *Actinobacteria*, *Bacteroidetes*. *Actinobacteria* was highly predominant in two of the low-yielding samples (i.e. > 85% of all sequence reads), while the fourth low-yielding sample (an unused ON sample) contained ~ 50% *Cyanobacteria*, followed by *Actinobacteria*, *Proteobacteria* and *Firmicutes* (Additional file 1: Table S5).

### Taxonomic richness and diversity by bedding type and status

**Fig. 2:**
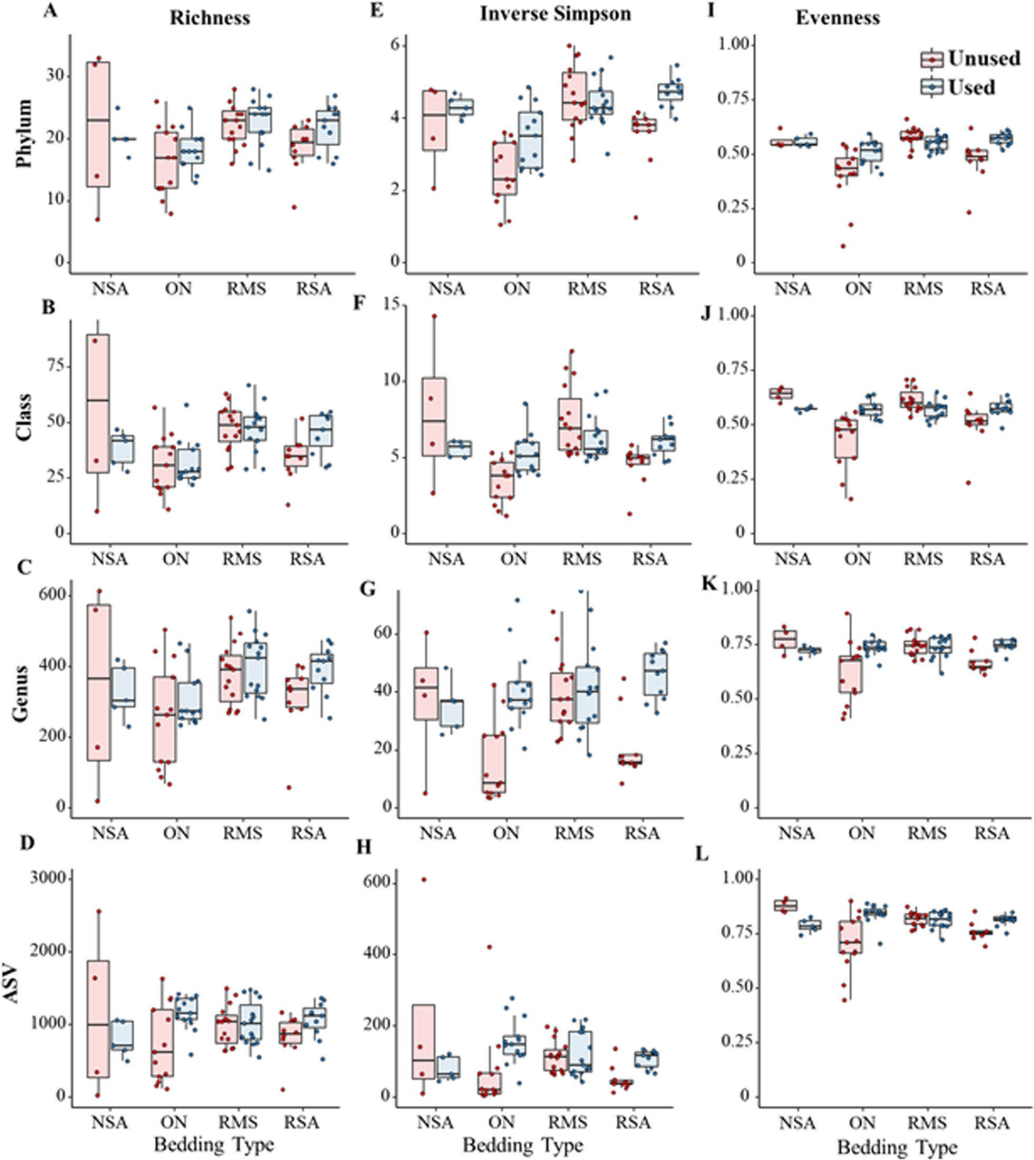
Box plots of alpha diversity indices (richness, inverse Simpson and Pielou’s evenness), by bedding type and status, at the phylum, class, genus, and ASV levels. Boxes represent the 25th to 75th percentile; horizontal line represents the median; and whiskers indicate 1.5× the interquartile range (IQR). *NSA* new sand, *ON* organic non-manure, *RMS* recycled manure solids, *RSA* recycled sand bedding type

To determine whether alpha diversity differed significantly by bedding type or status, we modeled richness, Inverse Simpson’s and Pieluo’s Evenness at the phylum, class, genus, and ASV levels using linear mixed-effects models. At the phylum, genus and ASV levels, bacterial community richness was higher in RMS samples compared to both NSA and RSA samples, while ON samples contained the lowest richness values (Fig. 2A–D). Multivariable modeling results indicated that bedding type was significantly associated with bacterial richness at the phylum (*P* = 0.01), class (*P* = 0.001) and genus (*P* = 0.05) but not ASV levels (*P* = 0.21, Additional file 1: Table S6). *Post-hoc* pairwise comparisons at the genus level indicated that the average richness was significantly lower in ON bedding compared to RMS. Similarly, the bacterial richness in used bedding samples was generally higher than in unused bedding samples at all of the analyzed taxonomic ranks, suggesting that used bedding contained more unique types of bacteria (Fig. 2A–D). However, this difference was only statistically significant at the class level, with unused samples containing 18 fewer classes of bacteria than used samples, on average (95% CI =  − 36 to − 1 classes, *P* = 0.04, Additional file 1: Table S6). The interaction between bedding type and status was not significantly associated with richness at the phylum (*P* = 0.52), class (*P* = 0.06) or genus levels (*P* = 0.67), but was at the ASV level (*P* = 0.03).

Inverse Simpson and Pielou’s Evenness indices showed similar trends to richness across bedding types, with RMS bedding generally containing higher diversity and evenness compared to NSA and RSA, with ON again exhibiting the lowest diversity and evenness across all taxonomic levels (Fig. 2E–L). Unlike with richness, however, the interaction between bedding type and status was significantly associated with Inverse Simpson’s and Pielou’s Evenness across all levels of the taxonomy (*P* < 0.01 for all model results, Additional file 1: Table S6), suggesting that changes in microbiome diversity and evenness during use by cows varied by bedding type, as suggested in Fig. 2. Used ON and RSA consistently contained higher diversity and evenness values than unused NSA, while the diversity and evenness in used RMS samples was not significantly different from unused NSA (Additional file 1: Table S6). The high level of variability in the richness and diversity of NSA samples may have influenced these findings (Fig. 2).

**Fig. 3:**
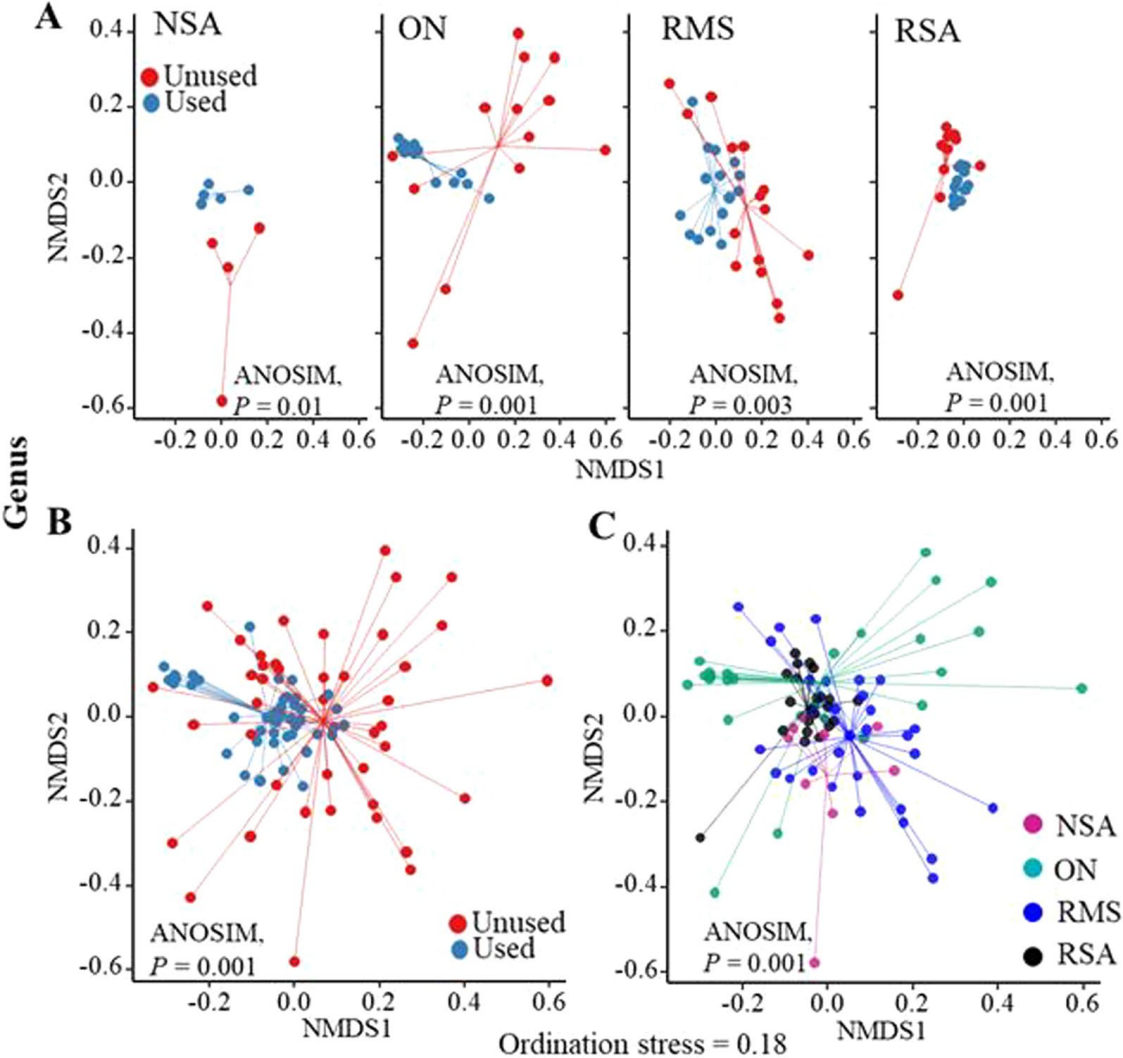
Non-metric multidimensional scaling (NMDS) ordination plots based on Bray–Curtis distances for **A** used versus unused status for each bedding type; **B** bedding status across all bedding types; and **C** all bedding types. *NSA* new sand, *ON* organic non-manure, *RMS* recycled manure solids, *RSA* recycled sand bedding type

To evaluate differences in overall bacterial composition, we generated NMDS ordination plots based on Bray–Curtis dissimilarity, which demonstrated clustering according to bedding type and status (Fig. 3A–C and Additional file 2: Fig. S1). The clustering of samples according to bedding status was more apparent in ON and NSA bedding types at every level of taxonomy. We observed that the overall bacterial community composition was impacted by both bedding type (PERMANOVA *P* = 0.001) and bedding status (PERMANOVA *P* = 0.001) as well as their interaction (PERMANOVA *P* = 0.001) at the phylum, class, genus, and ASV levels (Table 1). However, bedding status explained only 5.1–6.6% of the microbiome variation (depending on taxonomic level), whereas the bedding type explained 9.6–14.1% (Table 1). Similarly, the amount of dispersion in the ordination (i.e., dispersion of samples from the centroid of each group) varied significantly by bedding type (ANOVA *P* < 0.001) as well as bedding status (ANOVA *P* < 0.001), suggesting that the amount of variability in the microbial composition differed significantly between bedding types and status.

**Table 1:** PERMANOVA results for the effect of bedding type, status and their interaction on the microbial composition of bedding (*n*_permutations_ = 999)

Factor	Permutational multivariate analysis of variance (PERMANOVA)				
	Phylum	Class	Genus	ASV level	Potential mastitis pathogens*
Bedding type^a^	F = 3.47, *R*^2^ = 0.10, *P* = 0.001	F = 3.82, *R*^2^ = 0.11, *P* = 0.001	F = 6.09, *R*^2^ = 0.16, *P* = 0.001	F = 5.26, *R*^2^ = 0.15, *P* = 0.001	F = 4.49, *R*^2^ = 0.13, *P* = 0.001
Bedding status^b^	F = 7.18, *R*^2^ = 0.07, *P* = 0.001	F = 6.82, *R*^2^ = 0.06, *P* = 0.001	F = 7.85, *R*^2^ = 0.07, *P* = 0.001	F = 5.46, *R*^2^ = 0.05, *P* = 0.001	F = 10.07, *R*^2^ = 0.09, *P* = 0.001
Type × status	F = 4.05, *R*^2^ = 0.11, *P* = 0.001	F = 3.73, *R*^2^ = 0.10, *P* = 0.001	F = 2.85, *R*^2^ = 0.08, *P* = 0.001	F = 2.07, *R*^2^ = 0.06, *P* = 0.001	F = 1.91, *R*^2^ = 0.05, *P* = 0.001

*Ordination of potential mastitis pathogens was performed at the genus level

^a^Bedding type (*NSA* new sand, *ON* organic non-manure, *RMS* recycled manure solids, *RSA* recycled sand)

^b^Bedding status (unused and used)

### Differentially abundant taxa between unused and used bedding

**Fig. 4:**
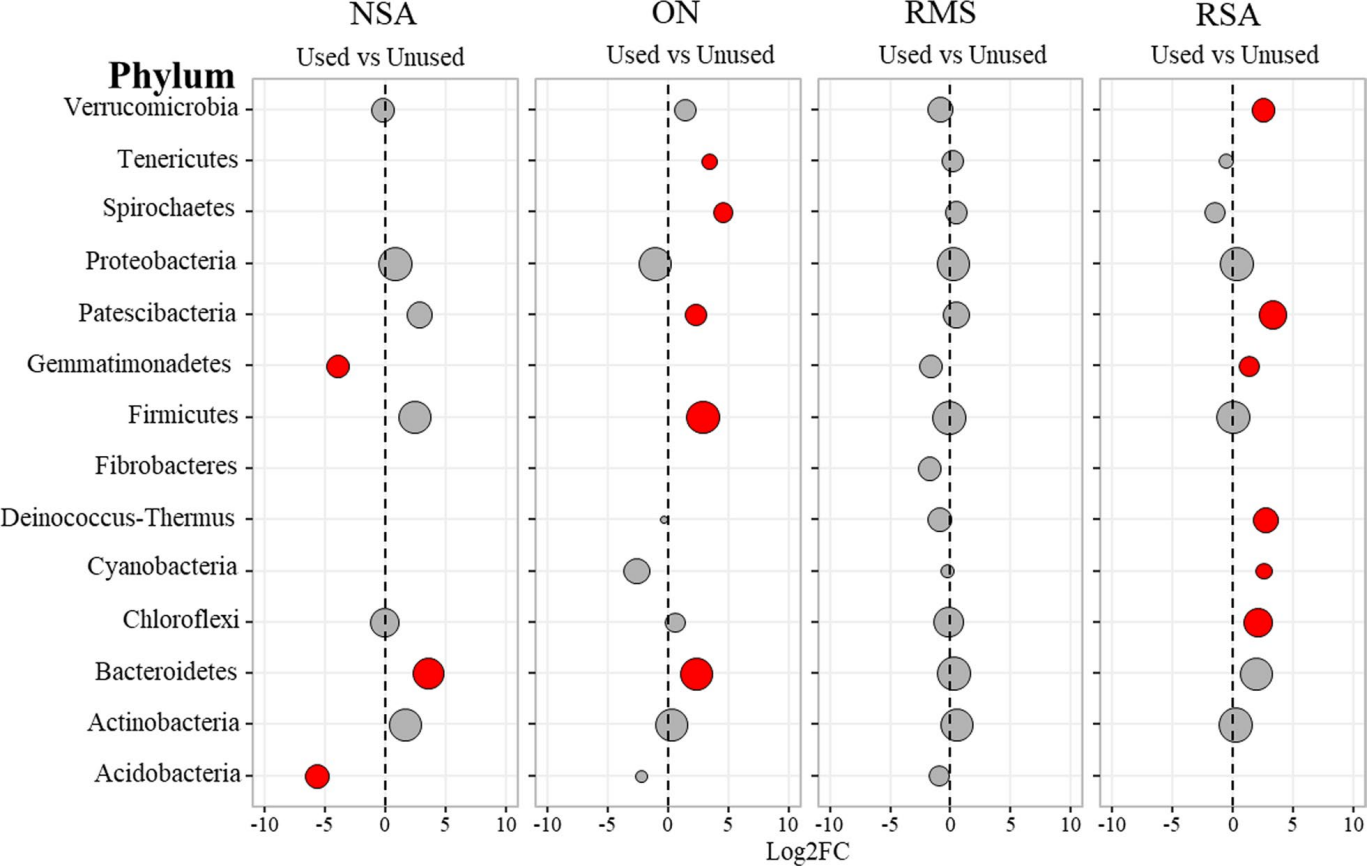
Log_2_-fold change (Log2FC) in abundance of phyla between used and unused bedding samples, separated by bedding type. Only phyla with an average abundance > 50th percentile within each bedding type are depicted. Red indicates phyla whose abundance was significantly different between used and unused bedding samples (i.e., adjusted *P* < 0.05). Circle diameter is proportional to the average abundance of each phylum across all samples within each bedding type. *NSA* new sand, *ON* organic non-manure, *RMS* recycled manure solids, *RSA* recycled sand bedding type

At the phylum level, 23 unique phyla exhibited statistically significant differences in abundance between used and unused bedding across all bedding types (Fig. 4). We restricted our visualizations to only those phyla whose average abundance was > 50th percentile within each bedding type, given that log-fold differences for very low-count taxa can be spuriously large. In RMS bedding samples, none of the phyla were significantly more or less abundant in used versus unused bedding, suggesting a relatively stable bacterial community at the phylum level. For ON and RSA bedding types, most or all of the differentially abundant phyla were more abundant in the used versus the unused samples. Bedding samples from NSA had overall lower phylum richness than the other sample types, with *Bacteroidetes* significantly more abundant in used versus unused samples; and *Gemmatimonadetes* and *Acidobacteria* more abundant in unused versus used samples. For a complete listing of logFC results at the phylum level, see Additional file 1: Table S7.

These trends were consistent at the class level, with NSA samples again containing much lower richness than the other sample types, with lower abundance in used versus unused samples for the classes *Gemmatimonadetes*, *Thermoleophilia* and Subgroup_6; and higher abundance for the *Bacteroidia* class (Additional file 1: Table S8 and Additional file 2: Fig. S2). As at the phylum level, RMS bedding samples did not contain any classes with significant differences in abundance between used and unused samples, suggesting that there were fewer differentially abundant taxa between used and unused RMS bedding compared to other bedding types. In contrast, RSA and ON bedding samples exhibited many phyla with differential abundance in used versus unused samples, the majority of which were more abundant in used versus unused samples (Additional file 1: Table S8 and Additional file 2: Fig. S2). For instance, among the differentially abundant taxa, *Bacteroidia* were significantly more abundant in used compared to unused NSA (mean expression = 11.6, LogFC = 3.5, *P* = 0.03) and ON (mean expression = 12.3, LogFC = 2.4, *P* = 0.02). Within ON bedding, members of the *Clostridia* class were much more abundant in used as compared to unused samples (mean expression = 11.1, LogFC = 4.0, *P* = 0.003), while the *Alphaproteobacteria* class was twofold lower (mean expression = 10.7, LogFC =  − 2.7,* P* = 0.02). *Thermoleophilia*, a class of bacteria responsible for biogeochemical cycling [28] had significantly lower abundance in used versus unused samples from both ON (mean expression = 3.3, LogFC =  − 2.4, *P* = 0.03) and NSA (mean expression = 6.8; LogFC =  − 6.2, *P* < 0.01).

At the genus level, 486 of the detected microbial genera exhibited statistically significant differential abundance between used and unused bedding, across all bedding types (Additional file 1: Table S9). Within NSA samples, 30 genera were significantly differentially abundant between used and unused samples, with 26 of those more abundant in used bedding and 4 more abundant in unused bedding. Within ON samples, 253 genera obtained statistical significance, with 174 more abundant in used samples and 79 more abundant in unused samples. Within RMS samples, 214 genera were found to differ significantly in abundance, with 99 more abundant in used samples and 115 more abundant in unused samples. Finally, in RSA samples, 165 genera had statistically significant differential counts based on bedding status, with 105 genera more abundant in used samples and 60 more abundant in unused samples. These differential abundance testing results suggest that both bedding status and bedding type influenced the presence and abundance of specific bacterial taxa.

### Presence of potential mastitis pathogens within 16S rRNA sequence data

**Fig. 5:**
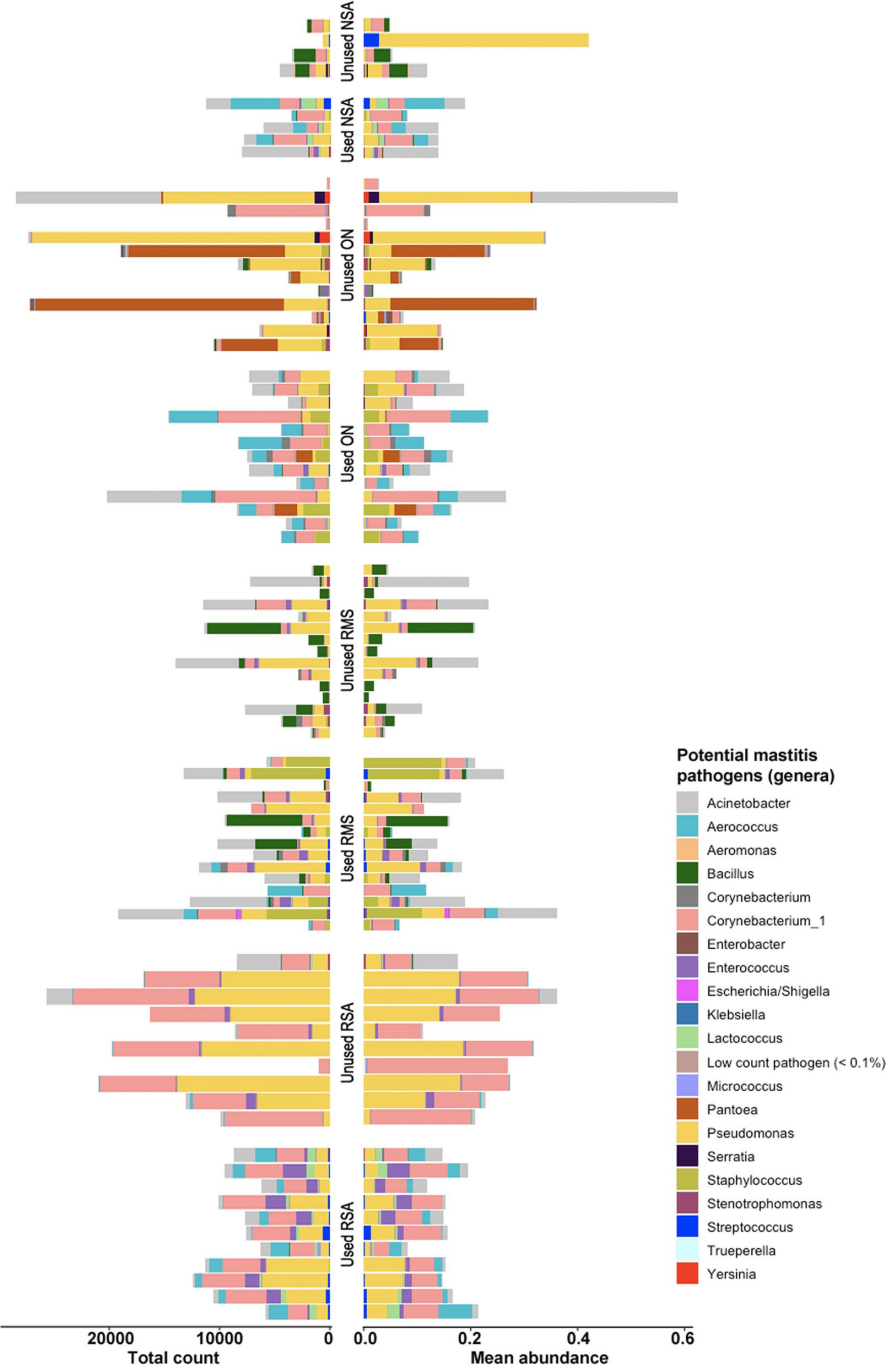
Barplot of total number of sequence reads (“total count”, left-hand side) and proportion of potential mastitis pathogens out of all genus-level counts, grouped by bedding status and type. Only genera with > 0.1% of the total genus-level counts are depicted as individual colors within the bars; those representing < 0.1% are grouped together as “low count pathogens”. *NSA* new sand, *ON* organic non-manure, *RMS* recycled manure solids, *RSA* recycled sand bedding type

In addition to looking at commensal bacteria, we also wanted to specifically evaluate bedding type and status for potential mastitis pathogens as identified by 16S rRNA sequencing (Additional file 1: Table S10). Although these potential mastitis pathogens were present in very low overall abundance (i.e., very low total sequence counts), we did detect several genera that could be considered potential mastitis causing pathogens (Additional file 1: Table S11). In general, most of the strict mastitis pathogens (e.g., *Staphylococcus*, *Streptococcus*), as well as other rare mastitis pathogens (e.g., *Acinetobacter*, *Pseudomonas,* and *Aerococcus*) were found in higher abundance in used RMS compared to unused RMS bedding (Fig. 5, Additional file 1: Table S11). Although low in abundance, *Escherichia/Shigella* increased in used ON, RMS and RSA bedding (Additional file 1: Table S12). Among the relatively rare mastitis pathogens, *Pseudomonas* and *Acinetobacter* were both prevalent and relatively abundant across all the bedding materials (Fig. 5), while *Corynebacterium* was also predominant in used and unused RSA and present in almost all other bedding types.

Based on differential abundance testing, several mastitis pathogens significantly differed in their abundance between unused and used bedding for each bedding type (Additional file 1: Table S12, Additional file 2: Fig. S3). For instance, *Staphylococcus* and *Streptococcus* had significantly higher abundance in used versus unused samples for both RMS (mean abundance = 3.92 and 3.0, logFC = 4.36 and 2.37, *P* = 0.005) and RSA (mean abundance = 1.84 and 4.76, logFC = 2.7 and 2.7, *P* = 0.003 and 0.002, respectively). Similarly, *Escherichia/Shigella* abundance was significantly higher in used versus unused ON (mean abundance = 1.73, logFC: 3.26, *P* < 0.001) and RMS bedding materials (mean abundance = 1.0, logFC: 3.67, *P* = 0.003). Similar results were observed for *Mycoplasma*, with significantly higher abundance in used compared to unused ON bedding type (mean abundance = 1.24, logFC: 2.19, *P* = 0.006). Some of the unused ON samples contained a preponderance of *Pantoae*, which was not identified in any of the other bedding types (Fig. 5); the prevalence of *Pantoae* was lower in used ON, however this was not statistically significant. *Aerococcus* was found to be significantly higher in used versus unused samples across all of the bedding types whereas *Lactococcus* was significantly more abundant in used versus unused NSA and RSA samples and was identified only very rarely in RMS (Additional file 2: Fig. S3). *Bacillus* was prevalent in both used and unused RMS and unused NSA, and was significantly lower in the used NSA samples; but was not prevalent in any other bedding type (Fig. 5).

### Presence and abundance of sequences from potential mastitis pathogens, by bedding type and status

The genus-level composition of potential mastitis pathogens varied by both bedding type (PERMANOVA *P* = 0.001) and status (PERMANOVA* P* = 0.001), with both factors explaining > 9% of the variation in the community structure of these potential pathogens (12.6% and 9.3%, respectively, Table 1, Additional file 2: Fig. S1). *Post-hoc* pairwise testing between bedding types indicated that the composition significantly differed between ON and RMS (*P* = 0.003), ON and NSA (*P* = 0.007), ON and RSA (*P* = 0.006), RMS and RSA (*P* = 0.003), NSA and RSA (*P* = 0.007), but not RMS and NSA (*P* = 0.14). However, there was also a significant interaction effect between bedding type and status (*P* = 0.002), suggesting that differences in the microbial composition between bedding matrices varied depending on whether the bedding was used or unused.

Associations between transformed potential mastitis pathogen counts based on 16S rRNA data (log_10_ scale) and bedding type, status and their interaction were evaluated using linear mixed models. Bedding type was not statistically significantly associated with 16S rRNA pathogen counts (*P* = 0.11), but bedding status was (*P* = 0.05). Specifically, these pathogen counts were higher in used versus unused bedding (average 4.2 vs. 4.0, 95% CI = 4.1–4.3 and 3.9–4.1, respectively). The interaction between bedding type and status was not significantly associated with the counts of potential mastitis pathogens at the genus level (*P* = 0.57).

### Relationship between 16S rRNA counts of potential bedding mastitis pathogens, *Staphylococcus* and *Streptococcus*, and bedding bacterial culture results

We performed Spearman correlation analysis to evaluate the relationship between 16S rRNA counts for all potential mastitis pathogens and total bacterial count (TBC) obtained from bedding aerobic culture (Table 2). There was a positive relationship between TBC and 16S rRNA pathogen counts for each bedding type and status except RSA (Additional file 2: Fig. S4). In unused RMS bedding, we found a strong positive correlation between TBC and 16S rRNA counts of potential mastitis pathogens (*ρ* = 0.74, *P* = 0.002, adjusted *P* = 0.03, Additional file 2: Fig. S4). Likewise, *Staphylococcus* exhibited a positive relationship between results obtained from 16S rRNA and culture, for both used NSA (*ρ* = 0.89, *P* = 0.04, adjusted *P* > 0.05) and used RMS (*ρ* = 0.68, *P* = 0.005, adjusted *P* = 0.1, Additional file 2: Fig. S4). *Streptococcus* -only counts obtained from 16S rRNA sequence and SSLO-CFU counts from bedding culture were also correlated for unused RMS (*ρ* = 0.58, *P* = 0.02, adjusted *P* > 0.05), as were SSLO 16S rRNA counts (*ρ* = 0.59, *P* = 0.02, adjusted *P* > 0.05, Additional file 2: Fig. S4). We did not find a significant correlation between 16S rRNA counts and culture-based results for any other sample types. For *Bacillus* and *Klebsiella*, culture-based results were largely invariable (i.e., each sample contained the same CFU/mL), and thus correlation analysis could not be performed (Additional file 1: Table S1). *Prototheca* was not identified in any of the samples based on culture.

**Table 2:** Spearman correlation coefficients (*ρ)* between culture-based bacterial counts and 16S rRNA based bacterial counts

		16S rRNA bacterial count															
		Potential mastitis pathogens count				*Staphylococcus* 16S rRNA counts				*Streptococcus* 16S rRNA counts				*Streptococcus, Aerococcus, Enterococcus and Lactococcus* *16S rRNA counts*			
		NSA	ON	RMS	RSA	NSA	ON	RMS	RSA	NSA	ON	RMS	RSA	NSA	ON	RMS	RSA
Bacterial Bedding Status Group^¥^																	
TBC^a^	Unused	*ρ* = 0.8, Unadj *P* = 0.33 (ns)	*ρ* = 0.54, Unadj *P* = 0.056 (ns)	*ρ* = 0.74, Unadj *P* = 0.002* (Adj *P* = 0.03)*	*ρ* = − 0.26, Unadj *P* = 0.47 (ns)												
	Used	*ρ* = 0.5, Unadj *P* = 0.45 (ns)	*ρ* = 0.46, Unadj *P* = 0.12 (ns)	*ρ* = 0.23, Unadj *P* = 0.4 (ns)	*ρ* = − 0.28, Unadj *P* = 0.4 (ns)												
* Staph*^b^	Unused					*ρ* = 0.26, Unadj *P* = 0.74 (ns)	*ρ* = 0.26, Unadj *P* = 0.39 (ns)	*ρ* = 0.29, Unadj *P* = 0.29 (ns)	*ρ* = 0.054, Unadj *P* = 0.88 (ns)								
	Used					*ρ* = 0.89, Unadj *P* = 0.041* (ns)	*ρ* = 0.51, Unadj *P* = 0.07 (ns)	*ρ* = 0.68, Unadj *P* = 0.005* (Adj *P* = 0.1)	*ρ* = − 0.021, Unadj *P* = 0.95 (ns)								
* SSLO*^c^	Unused									*ρ* = − 0.4, Unadj *P* = 0.75 (ns)	*ρ* = − 0.28, Unadj *P* = 0.36 (ns)	*ρ* = 0.58, Unadj *P* = 0.023* (ns)	*ρ* = 0.25, Unadj *P* = 0.48 (ns)	*ρ* = − 0.6 Unadj *P* = 0.42 (ns)	*ρ* = − 0.2, Unadj *P* = 0.54 (ns)	*ρ* = 0.59, Unadj *P* = 0.021* (ns)	*ρ* = 0.43, Unadj *P* = 0.22 (ns)
	Used									*ρ* = − 0.1, Unadj *P* = 0.87 (ns)	*ρ* = 0.31, Unadj *P* = 0.3 (ns)	*ρ* = − 0.17, Unadj *P* = 0.55 (ns)	*ρ* = 0.35, Unadj *P* = 0.29 (ns)	*ρ* = − 0.36, Unadj *P* = 0.55 (ns)	*ρ* = 0.18, Unadj *P* = 0.55 (ns)	*ρ* = 0.34, Unadj *P* = 0.22 (ns)	*ρ* = 0.19, Unadj *P* = 0.57 (ns)

The correlation was considered statistically significant at *P* < 0.05, and correlations were considered (±) strong when *ρ* ≥ 0.40 and (±) moderate between *ρ* ≥ 0.39 and ≥ 0.20

*NSA* new sand, *ON* organic non-manure, *RMS* recycled manure solids, *RSA* recycled sand bedding type

(ns), non-significant adjusted *P* value (i.e., *P* > 0.05), using Bonferroni adjustment for multiple comparisons)

^¥^Culture-based counts were modeled as log_10_ CFU/ml

^a^*TBC* total bacterial count

^b^*Staphylococcus* spp

^c^*Streptococcus and Streptococcus like organisms (SSLO)*

## Discussion

### The microbiome of unused bedding differs significantly by bedding material, and use by cows differentially alters this microbiome

Our results showed that the four evaluated bedding materials contained bacterial communities with significantly different structure and diversity, which was not surprising given the differing physico-chemical properties of these materials [6, 13]. In general, RMS was found to have greater microbiome richness, diversity and evenness at every taxonomic level compared to other bedding types, but there was no significant difference between unused and used RMS (Fig. 2). This result could indicate that the recycling process itself does not significantly decrease the number of unique types of bacteria in the bedding material, and does not significantly alter the relative distribution of the organisms in relation to each other. However, it is important to note that the 16S rRNA sequencing approach captures both live and dead bacteria, and thus these results cannot be used to make inferences about the viable portion of the microbial community; in other words, our results may have included remnant bacterial DNA that carried over from the recycling process. While previous studies have reported significant bacterial reductions during the manure recycling process using culture- and 16S rRNA based analyses, these studies focused specifically on potential pathogens, not the entire microbial community [6, 27], which may account for the discrepant findings.

Unlike RMS samples, RSA and ON samples exhibited clear increases in microbial richness, diversity and evenness when comparing used versus unused bedding (Fig. 2). This suggests that use by cows introduces new microbes to the microbiome of the bedding material. Furthermore, overall composition of the microbiome shifted significantly between used and unused bedding of all types (Fig. 3), indicating that use by cows and exposure to the dairy environment significantly alters the microbiome of all bedding materials, even when this bedding has high microbial diversity and a relatively stable microbiome, as in the case of RMS. The use of bedding by cows includes not only contact between the cow’s skin and the bedding, but also contamination of the bedding with urine and feces, which would introduce not only new bacteria into the bedding, but also novel substrates and physico-chemical conditions that could support differential growth or reduction of existing bacterial taxa.

The common impact of the cow microbiome may have also been reflected in a consistent increase in abundance during use, regardless of bedding type; such genera included *Marinobacter*, *Aerococcus*, *Confluentibacter*, and *Ornithobacterium* (Additional file 1: Table S9). Increase in other bacterial taxa were specific to certain types of bedding materials. For example, *Staphylococcus* was found in higher abundance in used bedding of all types except NSA; *Escherichia* in used ON and RMS; *Streptococcus* in used RSA and RMS; and *Mycoplasma* in used ON. These results indicate that both bedding status and bedding type play a role in the growth of various bacterial taxa during use by cows. Conversely, for some bacteria, use by cows was associated with a decrease in abundance. For example, *Pantoea* was found in high prevalence and abundance in unused ON samples, but then decreased significantly in used samples.

The common impact of the cow and farm environment on the bedding was also demonstrated by the lower beta-dispersion in the used versus unused samples across all bedding types except RMS. Given the geographic dispersion of the farms in this study, it is likely that the unused bedding materials were sourced from different suppliers, which likely explains the relatively high within-type heterogeneity of the unused bedding samples, particularly the NSA and ON samples. Additionally, the ON samples were sourced from a variety of raw materials including wood shavings, sawdust, rice hulls and paper, which likely also contributed to the high within-type heterogeneity of the unused ON samples. However, once the bedding was used by dairy cows, the heterogeneity reduced, likely due to exposure to cow feces, urine and skin, some of which have been shown to contain a core microbiome that is common across most dairy cows [29]. In effect, the cow microbiome becomes a “regularizing” factor that equilibrates the bedding microbiome as it is used. Based on our results, we conclude that different bedding types harbor differential microbiome profiles prior to use, but that ultimately the exposure to cows and the farm environment exerts a common influence on the in situ bedding microbiome, resulting in a significant shift in the bedding microbiome profile. The specific temporal and microbial ecological dynamics of this shift likely vary by bedding type and probably depend largely on the initial microbiome composition of each bedding lot. In any case, however, these dynamics may play a role in the differential influence that bedding type can have on prevalence of intramammary infection in late lactation dairy cows [26] and udder hygiene [6]. However, the existing literature on bedding and mastitis and/or udder health outcomes is mixed, with some studies finding no such associations [30]. This ambiguity could be driven by numerous potential confounding factors, including heterogeneity amongst the bedding materials used within a given bedding type, as well as variability in bedding management protocols between dairies. Further investigation into this question is warranted, especially given our findings that the microbiome differed significantly between bedding types, but that nearly all of the bedding samples exhibited a consistent shift during use by dairy cows, even across the diverse farms that comprised this study. While further research is needed, the fact that diverse bedding types all experienced a similar shift may represent an interventional opportunity for improved udder health in many herds. More research is needed to understand whether the dynamics of the microbiome shift during cow use are associated with udder health, mastitis epidemiology, or other important health and production outcomes on dairy farms.

### Low levels of DNA from potential mastitis pathogens were present in most bedding samples, with some differences between bedding types

We detected potential mastitis pathogens in most samples, however at very low relative abundance and with taxonomic resolution mostly to the genus level (Fig. 5). We observed significant differences in the composition and prevalence of genus-level taxa of potential mastitis pathogens across sample types (Fig. 5 and Additional file 1: Table S11), suggesting that different bedding matrices may support the presence of different potential mastitis pathogens. Though some studies have not observed significant associations between bacterial load, pH or dry matter and abundance of pathogens on the teat epithelium [31], others have reported epidemiological associations between bedding type and mastitis outcomes [23], and many of the previous investigations have focused on the differing physicochemical properties of the bedding material [32, 33]. Our findings support these interpretations by demonstrating that different bedding matrices support differential presence and abundance of genera that contain potential mastitis pathogens. However, not all of the differentially abundant bacteria are equally likely to cause mastitis, and each has a unique epidemiology within dairy herds. Our analysis treated each potential pathogen with equal weight, and thus must be interpreted cautiously, especially considering that many of the bacterial taxa on our list are very uncommon causes of mastitis [34] (Additional file 1: Table S10).

### Some potential mastitis pathogens were more abundant in used versus unused bedding, with highest levels in used RMS

We observed that many potential pathogenic genera were more abundant in used versus unused samples, across all bedding types (Additional file 1: Table S12). This again supports the hypothesis that exposure to both the cow and farm environment increases the likelihood that bedding material becomes contaminated with potential mastitis pathogens from these sources. This dynamic was most evident in the used versus unused NSA samples (Additional file 1: Table S11), which was expected given that these samples had no previous exposure to dairy cows. However, even in the case of RMS, we observed a significant increase in *Streptococcus, Staphylococcus, Escherichia/Shigella* and *Aerococcus* genera in used bedding, suggesting that even the high microbial diversity and biomass present in RMS was not enough to obscure the signal of contaminating mastitis pathogens in used samples. Indeed, used RMS samples contained the highest counts of *Staphylococcus* out of all sample types (Additional file 1: Table S12), and used RMS samples were the only ones in which we detected both *Staphylococcus chromogenes*, which is also considered a cow-adapted bacteria (Additional file 1: Table S3). While 16S rRNA data are typically reported at the genus level or higher, the use of ASVs does allow for species-level differentiation for some sections of the 16S rRNA taxonomy, depending on nucleotide-level variability within the relevant taxa. In these cases, identification of species is highly specific, which is one of the primary benefits of using ASVs [35]. Therefore, we can be confident that these species-level identifications within the used RMS samples are valid. However, the lack of species-level identification in other samples could be a potential false negative finding, particularly given the low classification rate at the species level, which is common to all 16S rRNA studies including those that use ASVs (Additional file 1: Table S2). Unfortunately, it is difficult to compare our species-level 16S rRNA results to previous bedding and udder microbiome studies because the use of ASVs for classification is a relatively recent advancement, and therefore existing studies report only at the genus level or higher based on non-ASV approaches. Previous culture-based studies have reported low prevalence of *S. chromogenes* in environmental samples taken from dairies [36], but the vast majority of results were obtained from udder or milk samples and thus relatively little is known about the extra-mammary ecology of this important bacteria [37]. Therefore, our detection of DNA from *Staphylococcus chromogenes* within bedding samples is difficult to contextualize, and warrants closer study. Previous studies have reported that RMS bedding supports the persistence and growth of some mastitis pathogens better than other bedding materials [26, 38], which is supported by our microbiome-focused results. However, the details of the recycling process can vary significantly between farms [39], and further research is needed to understand how different steps of the various recycling processes could impact the microbiome and presence/abundance of potential mastitis pathogens.

Although the counts of potential mastitis pathogens in our dataset were generally very low, we considered this to be a true reflection of the relative abundance of these taxa within each sample, as we observed a positive relationship between total BBC, *Staphylococcus* and *Streptococcus* sequence counts and bacterial culture data for most of the bedding types (Additional file 2: Fig. S4, Table 2). This correlation was particularly strong (and statistically significant) for RMS samples, again suggesting that this matrix occupies a unique position in the complex epidemiology of mastitis pathogens and bedding microbial ecology. Further research is needed to evaluate correlations between mastitis pathogen results obtained from culture-independent and culture-dependent approaches, as we found varying correlations depending on the pathogen and bedding type (Additional file 2: Fig. S4, Table 2). Additionally, future work should consider techniques that can more robustly differentiate species-level taxa, including more systematic use of MALDI-TOF for culture-based work and shotgun metagenomic sequencing for culture-independent workflows.

### Some bedding samples contained very low microbial biomass, which complicates interpretation of microbiome data

Some of the samples in this study, particularly those collected from unused NSA, yielded very low concentrations of total DNA and 16S qPCR copy number, suggesting very low microbial biomass. Previous studies have demonstrated that the physicochemical properties of these types of samples, such as very low organic matter or very low moisture levels, may not support rapid bacterial growth [9], and thus the low microbial biomass was expected. However, such low biomass samples require careful consideration in microbiome studies given the possibility of contamination from extraction kit reagents, especially PCR master mix and even molecular biology grade water [40–42], which can sometimes exceed the abundance and diversity of the resident microbiome [41]. To control for this, we included negative controls and used them to identify and remove likely contaminants from the sequence data [43, 44]. Despite these internal controls, it is important to note that cross-contamination could still explain some of the extreme variability in microbial composition of the data obtained from these samples, particularly within unused NSA samples (Figs. 2, 3, 5). Future bedding microbiome studies of low biomass samples such as sand should include extensive negative controls, including samples from collection buckets and gloves, which can be used to account for contamination that occurs during the sampling process. Additionally, sample collection strategies may need to be optimized specifically for these low biomass samples; fortunately, recommendations exist [45]***.*** Previous research has shown that larger volumes of low-biomass samples don’t necessarily lead to significantly increased DNA biomass [46], and therefore future efforts may yield more success by focusing on improved extraction methods [47].

### Comparison with previous descriptions of the bedding microbiome

While the literature regarding the microbiome of dairy bedding is scarce, previous investigations also show predominance of *Micrococcus*, *Arthrobacter*, *Staphylococcus*, *Bacillus*, *Corynebacterium*, *Microbacterium*, *Streptomyces*, *Acinetobacter*, *Proteus*, *Pantoea*, *Pseudomonas*, *Thermoactinomyces*, and *Saccharopolyspora* [48]. However, some previous results are discordant with our observations. For example, *Aerococcaceae* have been characterized as a dominant and prevalent taxon within bedding [16], but our results show that this taxon only appears in substantial abundance in used bedding material, suggesting that *Aerococcus* growth is an outcome of bedding use by cows, and not necessarily a resident bacteria in unused bedding. Ambiguous results such as these emphasize the need to carefully document the status, type and physicochemical properties of the bedding being analyzed; and to report these details so that microbiome results can be reliably and robustly compared across studies. Such challenges are not unique to bedding microbiome research, and numerous efforts are underway to promote standardized collection and reporting of such metadata [49, 50].

### Study limitations and strengths

Many of the limitations of our study are common to microbiome studies, including well-documented biases and limitations in detection of some taxa. To provide a measurement of these potential biases, we utilized ZymoBIOMICS Spike-in Control II and aligned the resulting sequence data to a database containing only the three bacteria contained within the mock sample, i.e., *Truepera radiovictrix*, *Imtechella halotolerans*, and *Allobacillus halotolerans*). Classifying all of the reads from the mock community dataset to the SILVA database identified *T. radiovictrix* as the most abundant organism and *I. halotolerans* as the third-most abundant organism (Additional file 1: Table S13) as expected based on the true composition of the mock community, which contains a predominance of *T. radiovictrix* and tenfold lower abundance of *I. halotolerans*. The distribution of these two bacteria in our mock sample, however, was not precisely tenfold different, likely due to the known lysis resistance of *Truepera*, which reduced the efficiency of the DNA extraction. Furthermore, *Truepera’s* high GC content challenges primer-based assays and is a well-known issue [51]. In addition, we did not identify *A. halotolerans* when aligning the sequence data for the mock sample to the SILVA database, likely due to the lack of species-level ASV resolution for *Bacillaceae* in the V3/V4 region. To circumvent this limitation, we aligned the sequence data from the mock community to *only* the 16S rRNA sequences of the three expected bacteria, which resulted in detection of all three taxa with *A. halotolerans* comprising ~ 6% of the reads. Together, our positive control results suggest that hard-to-lyse and high-GC-content bacteria may be systematically underrepresented within the data, which is not uncommon for microbiome studies [38]. Furthermore, we were able to detect *Allobacillus halotolerans* in the positive control sequence data, suggesting that our sequencing depth was sufficient to detect low-abundance taxa within the microbial communities.

The inability to classify sequences to the species level is a further well-documented limitation of 16S rRNA based analysis [52, 53]. While the V3–V4 hypervariable regions used in this study are very common and provide a comprehensive overview of most microbiomes [54], they may not be the optimal targets for identification of mastitis pathogens at the species level, which limits our ability to fully characterize potential pathogens [55]. Previous studies have reported that a 28 nucleotide-long region within the V1 hypervariable region has the most discriminatory power for differentiating *Staphylococcus aureus* from other coagulase negative *Staphylococcus* sp., and future studies may want to use this region if pathogen evaluation is the primary goal [56]. Additionally, future studies of the bedding microbiome should consider including multiple complementary approaches for more robust and comprehensive species-level identification, including shotgun metagenomics, MALDI-TOF-confirmed culture, and qPCR.

Finally, the inability to distinguish live versus dead bacteria is a limitation of the 16S rRNA based approach and may obfuscate any associations between the bedding microbiome and biological outcomes in dairy cows. Our findings provide some counterweight in this regard, as we identified a consistent positive correlation between genus-level counts of mastitis pathogen sequences and counts obtained from cultural bacteriology of these same pathogens from the same samples, suggesting that at least some of the DNA in the microbiome workflow originated from viable cells. Further studies are needed to confirm whether (and under what specific conditions) 16S rRNA-based counts correlate with culture-based results, as well as to differentiate DNA from viable versus non viable bacteria. The use of multiple complementary culture- independent and -dependent workflows is especially important in this regard, as their results will support improved understanding of whether bedding microbiome dynamics support pathogen persistence or transmission, and whether the bedding microbiome plays a role in mastitis etiology.

In addition to these limitations, our study contained several notable strengths, including the evaluation of multiple bedding materials across 44 farms located in ecologically diverse climates. The heterogeneity of this source farm population provides increased external validity of our findings compared to many bedding studies that were conducted on fewer or more homogeneous farm populations. However, it should also be noted that the distribution of bedding types represented in this study may not reflect the distribution of bedding used across the U.S. dairy farms. The inclusion of used and unused bedding samples was a strength in the study design, and highlighted the fact that the bedding microbiome experiences significant temporal shifts. This insight should be used to guide the design of future bedding microbiome studies, and emphasizes the importance of reporting detailed sample-level metadata for bedding samples. Finally, our use of negative control allowed us to differentiate contaminating from non-contaminating DNA, which is very germane for the low-biomass bedding samples we encountered in this work.

### Future research

Our study was limited to description and comparison of the microbiome of various bedding types and status. While we identified significant differences in the microbiome of different bedding materials, we were not able to connect these differences to important outcomes of udder health such as mastitis incidence or somatic cell count. Future studies that wish to evaluate associations between bedding and mastitis should consider integrating bedding microbiome analysis into their plans in order to account for the microbiome as either a confounder or a primary risk factor. Furthermore, our results support previous work suggesting that RMS is a complex bedding material, which may cause variable impacts on udder health and mastitis. The body of work on RMS and mastitis is somewhat ambiguous, potentially due to the wide variability in how RMS are produced [39]. Further research is needed to elucidate potential interactions between the manure solids recycling process, the microbiome and mastitis pathogens, and udder health outcomes.

## Conclusions

In the present study, we aimed to describe the microbiome of used and unused bedding samples representing a variety of commonly used materials. Our results demonstrated that different bedding materials harbored different microbiomes prior to use by cows; and that use by cows significantly shifted this microbiome. These differential microbiomes may explain some of the previous epidemiological associations reported between bedding material and mastitis outcomes, but further research is needed to test this hypothesis. We found that genera containing potential mastitis pathogens generally comprised a very small proportion of the overall microbial community; however, the counts of these genera correlated positively with culture-based results, suggesting that the sequence-based counts may represent biologically meaningful information. Samples obtained from RMS bedding exhibited different microbiome and potential pathogen dynamics than the other types of bedding, supporting previous findings that RMS may play a unique role in mastitis epidemiology and suggesting that the recycling process may need closer investigation. Overall, these results emphasize that the bedding microbiome deserves closer investigation, particularly with respect to its potential mechanistic role in explaining epidemiological associations between bedding management and mastitis outcomes in commercial dairy herds.

## Methods

### Farm description and sampling

This study used samples collected from commercial dairy herds across 10 states in the U.S., and was part of a larger study that evaluated bedding and mastitis epidemiology [26]. The intent to use these samples for microbiome analysis was conceived before samples were collected, but after funding for the larger study had been obtained. For the larger study, 80 herds were selected based on the following inclusion criteria: herd size > 200 cows; collaborative work with the University of Minnesota or a local Zoetis Quality Milk Specialist; and use of one of four common bedding types, described previously [26]. From the 80 enrolled herds, 44 were selected for inclusion in this microbiome analysis, with farms chosen based on availability of samples that had undergone fewer than two freeze–thaw cycles, as freeze thaw cycles were previously reported to introduce bias in microbiome studies [57, 58]. Further details on the study population can be found in [26].

### Bedding sample collection

Different dairy farms in the study utilized different bedding materials (with only one type used per farm), with the following types represented: new inorganic or new sand (NSA, N = 5, collected from WI, TX, CA and ID), recycled manure solids (RMS, N = 15, collected from NY, CA, ID, MN, WI and WA), other organic non-manure (ON, N = 13, collected from WI, MN, NY and WA), and recycled inorganic or recycled sand (RSA, N = 11, collected from NY, WI, IN, OR and MI). From each farm, ‘unused’ (ready-to-use) bedding was collected from the stockpile, while ‘used’ bedding was collected from stalls that were actively being used by dairy cows. Unused and used bedding samples (hereafter referred to as “bedding status”) were collected on the same day at each participating farm. For sampling, collectors from Zoetis Quality Milk Specialists followed a standardized collection protocol in which 20 handfuls of unused bedding materials from various sections of the unused bedding pile were placed into a disinfected bucket and mixed thoroughly. From that homogenized sample a subsample of approximately one litre was transferred into a resealable plastic bag, which was manually expressed to remove excess air, and then sealed. All the used bedding samples in this study were collected from freestall herds in the following manner: one handful of bedding material was collected from the top 5 cm of the back third of at least 20 stalls in the late-lactation pen, with care taken to avoid obvious manure pats during sampling. The 20 handfuls were placed into a bucket and the procedure followed the same protocol as described for unused bedding. The bucket was disinfected with chlorhexidine between each sampling and investigators used new gloves before handling each bedding sample. Samples were frozen at the time and location of collection (− 20 °C), and later shipped, on ice, to the Laboratory for Udder Health, University of Minnesota (St. Paul, MN). Upon arrival at the University of Minnesota (UMN), the bedding samples were immediately placed in − 80 °C for long-term storage after taking an aliquot of each bedding sample for aerobic bacterial culture.

### Bacterial culture of bedding samples

For bacterial culture, 50 mL of bedding material was sub-sampled, weighed and transferred to a sterile plastic bag (Whirl–Pak, Nasco, Fork Atkinson, WI) along with 250 mL of sterile water to create a 1:5 dilution. After the bedding-water mixture was homogenized, four different dilutions (1:5, 1:50, 1:500, and 1:5000) of the bedding suspension were made to inoculate onto Columbia CNA agar with 5% sheep blood (CNA) and MacConkey agar plates. Bedding cultures were incubated in aerobic conditions at 37 ± 2 °C for 42 to 48 h before reading colonies. Bacterial groups were identified using visual inspection and enumerated from the dilution plate with the optimal number of colonies (25 to 250 per plate). Representative isolates from each plate were further subjected to confirmation via matrix assisted laser desorption ionization-time of flight mass spectrometry (MALDI-TOF MS). Organisms belonging to the “*Streptococcus* and Streptococcus-like organisms” (SSLOs) were grouped together due to inability to differentiate the taxa based on visual inspection; these taxa comprise *Streptococcus*, *Enterococcus*, *Lactococcus* and *Aerococcus*. The counts from each bacterial group were summed to determine total bacterial count. These results have been published and the details are available [26].

### DNA extraction, library preparation and 16S rRNA gene sequencing

Bedding samples were removed from − 80 °C, thawed at − 20 °C and then room temperature and homogenized before DNA extraction. DNA was extracted using the DNeasy PowerSoil Pro Kit (Qiagen, Cat No. 47016, Hilden, Germany) following the manufacturer’s instructions. Briefly, bedding materials were weighed inside a biosafety cabinet using a sterile disposable spatula. Straw type bedding materials could not be weighed to the maximum capacity (0.25 g) due to volume constraints of the bead tubes (Additional file 1: Table S1). Lysis (CD1) buffer was added to the bead tubes after adding samples. The volume of the CD1 buffer varied depending on the sample type; most samples were processed with 800 µL of CD1 buffer, but the absorbency of sawdust and straw bedding materials necessitated 1200 µL of CD1 buffer to extract 600 µL for the subsequent steps of DNA extraction. After vortexing, bead tubes were processed on a Mini Bead-beater (Biospecproducts Cat. No. 1001, Bartlesville, OK, U.S.) at 2200 rpm for 20 s, which was repeated 3 times with an interval of 30 s in between rounds. Bead tubes were then centrifuged at 15,000 g for 1 min to precipitate the debris, and 600 µL of supernatant was transferred to the rotor adapter of QIAcube Connect (Qiagen, Cat No. 9002864, Hilden, Germany) for DNA extraction. All samples were processed with Inhibitor Removal Technology (IRT) to eliminate inhibitors. Finally, extracted DNA was eluted in a 50 µL elution buffer. DNA concentration was measured with Qubit 4 Fluorometer (ThermoFisher Scientific, Cat No. Q33226, Hercules, CA, U.S.) and quality was checked with Tapestation genomic screen tape (Agilent Technologies, Palo Alto, CA). In addition to the bedding samples, we extracted DNA from 100 µL of ZymoBIOMICS Spike-in Control II (Zymoresearch, Cat No. D6321, Irvine, CA, U.S.) as a positive control in the same way along with the samples except 700 µL of CD1 buffer was added in the bead beating tube. We also included molecular biology grade water (AccuGENE^™^ Water, Cat No. BE51200), as negative control (NTC1) or amplification blank.

The 16S rRNA gene copy number in each sample was measured using qPCR in order to begin the library preparation with an approximately equal amount of bacterial DNA across samples. For sequencing, the target copy number threshold was set at 167,000 molecules/uL. For 16S rRNA library preparation, samples were amplified using a dual-indexing 16S rRNA Illumina primer set (Forward primer: 5′-TCGTCGGCAGCGTCAGATGTGTATAAGAGACAGCCTACGGGAGGCAGCAG- 3′ and Reverse primer: 5′-GTCTCGTGGGCTCGGAGATGTGTATAAGAGACAGGGACTACHVGGGTWTCTAAT- 5′-) specific to the V3–V4 region [59]. PCR products were quantified using a PicoGreen dsDNA assay kit (Life Technologies, Carlsbad, CA), normalized and multiplexed in equimolar amounts. The sample pool was spiked with 15% PhiX and sequencing was performed at the University of Minnesota Genomics Center (UMGC) using Illumina’s v3 cluster chemistry (2x300 bp paired-end reads) on the MiSeq platform (Illumina Inc., San Diego, CA).

### Sequencing data processing

Amplicon primers were removed from the 5′ and 3′ ends of forward and reverse reads using cutadapt [60]. Trimmed sequence reads were processed using the DADA2 (Divisive Amplicon Denoising Algorithm) pipeline, version 1.12 [35]. The *filterAndTrim* function was used for further quality filtering. Forward and reverse reads were truncated to 250 and 220 base pairs, phiX reads were discarded as were reads with a maximum expected error rate greater than 3. Filtered sequence reads were then used as input to the *learnErrors* function for error-rate estimation. The error-rate matrix was used as input to the *dada* function for denoising (i.e., read error correction). Error corrected forward and reverse reads were merged into contigs using the *mergePairs* function. Amplicon sequence variant table (ASV) table was generated after removing chimeric contigs using the *removeChimera* function. The *assignTaxonomy* function was used for taxonomic assignment of ASVs using the SILVA reference database by native implementation of the naive Bayesian classifier method [61]. The *addSpecies* function was used to assign species-level labels to annotated ASVs. The positive control sample sequenced in this study was aligned to both the SILVA database and a ZymoBIOMICS sequence database (https://s3.amazonaws.com/zymo-files/BioPool/D6321.refseq.zip) containing reference sequences for each mock bacterium, using the same procedures described above. The abundance matrix and taxonomy table produced by the DADA2 pipeline was imported into phyloseq for microbiome analysis and visualization [62]. Contaminating ASVs were identified using the frequency method implemented in decontam, and removed from further analysis [43].

### Sequencing depth

To evaluate potential sequencing bias by bedding type and status, the number of raw reads generated for each sample were compared using generalized linear modeling as implemented in the *glm* function. Model results and confidence intervals for each variable (i.e., bedding type and status) were extracted using the *summary* and *confint* functions. The significance of each variable was evaluated using the *anova* function.

### Analysis of microbial community structure by bedding type and status

Alpha diversity was measured from the decontaminated abundance matrix by computing richness, Inverse Simpson, and Pielou's evenness [63] indices. Richness and diversity were computed using the *estimate_richness* function in phyloseq [62]. Evenness was computed using the *evenness* function in the microbiome package (https://microbiome.github.io/). Alpha diversity was measured following aggregation of ASVs to the phylum, class, family, and genus levels using the “tax_glom” function in phyloseq. The association between each alpha diversity metric (i.e., richness, Inverse Simpson’s and Pielou’s evenness) and bedding type and bedding status and their interaction (explanatory variables) were analyzed using linear mixed-effect models as implemented in the *lme* function [64]. Farm identity was included as a random effect. The significance of each explanatory variable in improving model fit was assessed by comparing the full model with the reduced model using the ANOVA function in R, with a significance level of *P* < 0.05*.* For variables that significantly improved model fit, post hoc pairwise comparisons of bedding type and status were performed using the *lsmeans* function.

The overall pattern of microbial community composition (beta-diversity) across all bedding types and status was visualized using non-metric multidimensional scaling (NMDS) plots from a Bray–Curtis distance matrix, using the vegdist function of vegan in R. To test for significant associations between bedding type and status on the ordination, permutational multivariate analysis of variance (PERMANOVA) was used via the *adonis* function of vegan in R. The R^2^ value was used to estimate the relative effect size (i.e., percent of community structure variation explained by each explanatory variable), and the corresponding *P* value was used to determine the statistical significance of this value. If a significant result (*P* < 0.05) was observed, post hoc pairwise comparisons of bedding types and status were conducted using the *pairwise.adonis* function. The *betadisper* function was used to calculate the homogeneity of multivariate dispersions by bedding type or status (i.e. deviation from centroids), with analysis of variance (ANOVA) testing to determine if the dispersion differed significantly between bedding types and status. Differences in microbial community structure between bedding types and status were also tested using analysis of similarities (ANOSIM) with the anosim function in vegan.

### Differential abundance testing to identify differences in relative abundance of bacterial taxa between bedding types and status

To identify sequence features that were differentially abundant between unused and used bedding for each bedding type, we performed multivariate zero-inflated Gaussian mixture models as implemented in the fitZig function in *metagenomeSeq* [65], following aggregation of sequence features to the phylum, class, and genus levels. Sequence features with fewer than 5 total read counts were discarded. The filtered abundance matrices from each aggregated matrix were normalized using the *cumNorm* function in *metagenomeSeq*, using a default normalization factor of 0.5 [65]. Farm identity was included as a random effect. Pairwise comparisons of taxa abundance between bedding status and type were calculated using the *makeConstrast* function in limma [66] with Benjamini-Hochberg (“BH”) correction for multiple testing. Log_2_-fold change (LogFC) and mean expression values between comparison groups for each taxon with BH adjusted *P* values were derived from the models using the *topTable* function in limma. Taxa with a mean expression value above the 50th percentile within the relevant comparison groups were selected and visualized in a stratified manner for each bedding type.

### Sequenced-based evaluation of potential mastitis pathogens, with comparisons between bedding types and status

To evaluate the presence of potential mastitis pathogens within the 16S rRNA data, we first identified a list of potential pathogens (Additional file 1: Table S10) [67] and then subsetted the genus-level count matrix to only include the listed pathogen candidates. Beta-diversity analysis and differential abundance testing were performed on this subsetted count matrix as described above for the complete count matrix. Briefly, ordination was performed, followed by PERMANOVA testing to assess the effect of bedding status and type. Differential abundance (logFC) was evaluated for each potential pathogen, comparing used and unused bedding by type. Associations between the normalized genus-level sequence counts of the potential pathogens and bedding type and status were determined using linear mixed models, using the same modeling approach as described above for alpha diversity comparisons.

### Correlation between 16S rRNA based sequence counts of potential mastitis pathogens and culture-based bacterial counts

To test whether the 16S rRNA sequence data correlated with culture-based data, we performed Spearman correlation analysis between the genus-level log_10_-transformed counts of potential pathogens from the 16S rRNA sequence data and culture-based counts measured as log_10_ colony-forming units per mL, or CFU/mL obtained from aerobic culture of the same bedding samples. From the companion culture-based study [26], we obtained culture results for *Staphylococcus* spp., *Streptococcus* spp. and *Streptococcus*-like organisms (SSLO, which included *Streptococcus*, *Enterococcus*, *Lactococcus* and *Aerococcus*), coliforms, *Klebsiella* spp., non-coliform gram-negatives, *Bacillus* spp., *Prototheca*, and all bacteria (i.e., total bacterial count or TBC). When possible, we compared these culture-based results to the 16S rRNA results using correlation analysis at the genus level. This analysis was not performed for coliforms and non-coliform gram-negatives due to an inability to extract the appropriate taxa from the 16S rRNA taxonomy. To investigate correlation specifically between *Streptococcus* 16S rRNA counts and culture results, we compared *Streptococcus*-only 16S rRNA counts with culture-based CFU counts of SSLO’s; and we compared 16S rRNA counts for *Streptococcus*, *Enterococcus*, *Lactococcus* and *Aerococcus* (combined) with culture-based SSLO CFU counts. We also performed correlation analysis on TBC and the log_10_-transformed sequence counts from all potential mastitis pathogens at the genus level. Results of all correlation analyses were visualized using scatter plots. Bonferroni correction was used to account for multiple comparisons, and adjusted *P* values were reported along with unadjusted *P* values (Stata/MP 17.0, StataCorp LLC, College Station, TX, USA).

All statistical analysis was performed in R (version 3.6.1, https://www.r-project.org/), and results were visualized using ggplot2 [68]. For all statistical analysis, unless otherwise indicated, significance was determined as *P* < 0.05.

## Supplementary Information

**Additional file 1: Table S1.** Metadata file for all samples included in this study. **Table S2.** ASVs assigned to each taxonomic level. **Table S3.** Read counts and mean abundance of assigned species stratified by sample ID. **Table S4.** Proportion of phylum-level counts, by bedding type and status. **Table S5.** Proportion of phylum-level counts, for the four non-outlier low-yielding bedding samples. **Table S6.** Modeling results for associations between bedding status and type, and alpha diversity metrics (LMM output). **Table S7.** Results from phylum-level differential abundance testing of used versus unused samples, by bedding type. **Table S8.** Results from class-level differential abundance testing of used versus unused samples, by bedding type. **Table S9.** Results from genus-level differential abundance testing of used versus unused samples, by bedding type. **Table S10.** List of potential mastitis pathogens considered in the analysis of 16S rRNA sequence data. **Table S11.** Proportion of genus-level counts for genera that contain potential mastitis pathogens, by bedding type and status. **Table S12.** Results from genus-level differential abundance testing of taxa that contain potential mastitis pathogens, comparing used versus unused samples, by bedding type. **Table S13.** Read counts and proportion of genus-level counts for the mock community and negative control samples.

**Additional file 2: Fig. S1.** Non-metric multidimensional scaling (NMDS) ordination plots based on Bray–Curtis distances for (**A**) used versus (**B**) unused status for each bedding type, at the phylum, class and ASV level; and for potential mastitis pathogens at the genus level. NSA—new sand, ON—organic non-manure, RMS—recycled manure solids and RSA—recycled sand bedding type. **Fig. S2.** Log_2_-fold change (Log2FC) in abundance of classes between used and unused bedding samples, separated by bedding type. Only classes with an average abundance >50th percentile within each bedding type are depicted. Red indicates classes whose abundance was significantly different between used and unused bedding samples (i.e., adjusted *P *< 0.05). Circle diameter is proportional to the average abundance of each genus across all samples within each bedding type. NSA—new sand, ON—organic non-manure, RMS—recycled manure solids and RSA—recycled sand bedding type. **Fig. S3.** Log_2_-fold change (Log2FC) in abundance of genera that contain potential mastitis pathogens, comparing used and unused bedding samples, separated by bedding type. Only genera with an average abundance >50th percentile within each bedding type are depicted. Red indicates genera whose abundance was significantly different between used and unused bedding samples (i.e., adjusted *P *< 0.05). Circle diameter is proportional to the average abundance of each genus across all samples within each bedding type. NSA—new sand, ON—organic non-manure, RMS—recycled manure solids and RSA—recycled sand bedding type. **Fig. S4.** Scatter plots of 16S rRNA gene counts and culture results obtained from the same bedding samples, for: total bacteria (panels **A**–**D**, TBC); *Staphylococcus* (panels **E**–**H**); 16S rRNA gene counts for *Streptococcus* and culture-based *Streptococcus *and *Streptococcus* like organisms (SSLO) counts (panels **I**–**L**); 16S rRNA gene counts for *Streptococcus*, *Aerococcus*, *Enterococcus* and *Lactococcus* and culture-based *Streptococcus* and *Streptococcus* like organisms (SSLO) counts (panels **M**–**P**); *Bacillus *(panels **Q**–**T**), and *Klebsiella* (panels **U**–**X**). NSA—new sand, ON—organic non-manure, RMS—recycled manure solids and RSA—recycled sand bedding type.

## Data Availability

The raw sequence data generated during this study are available in the NCBI repository under BioProject ID: PRJNA780498. Metadata and supplementary data are provided with the manuscript.
