# Supplementary figures and images for: The microbiome of common bedding materials before and after use on commercial dairy farms
Source: Anim Microbiome. 2022 Mar 7;4:18. doi: 10.1186/s42523-022-00171-2 (PMC8900318; doi:10.1186/s42523-022-00171-2)

Figure S1

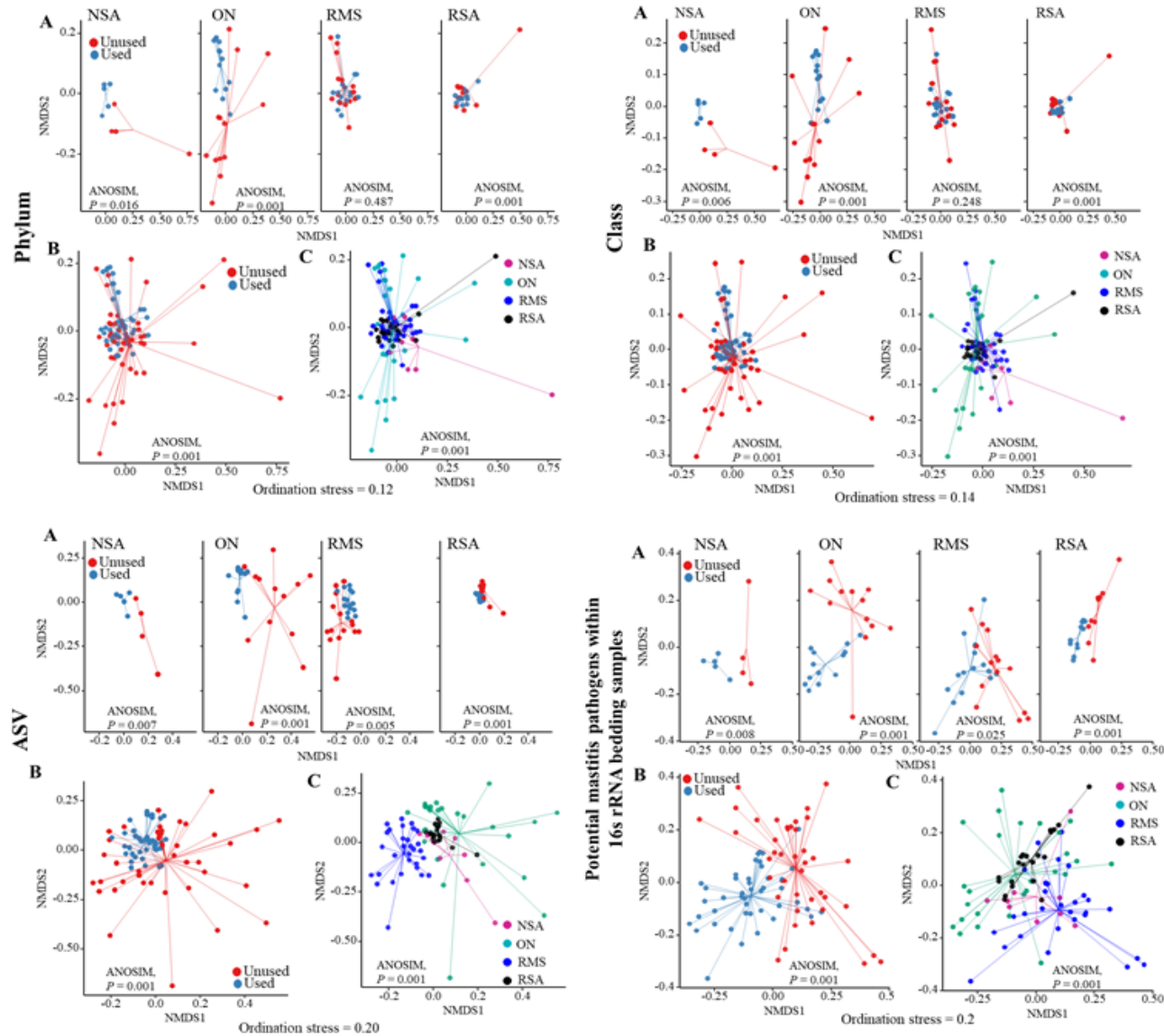

**Figure S2.**

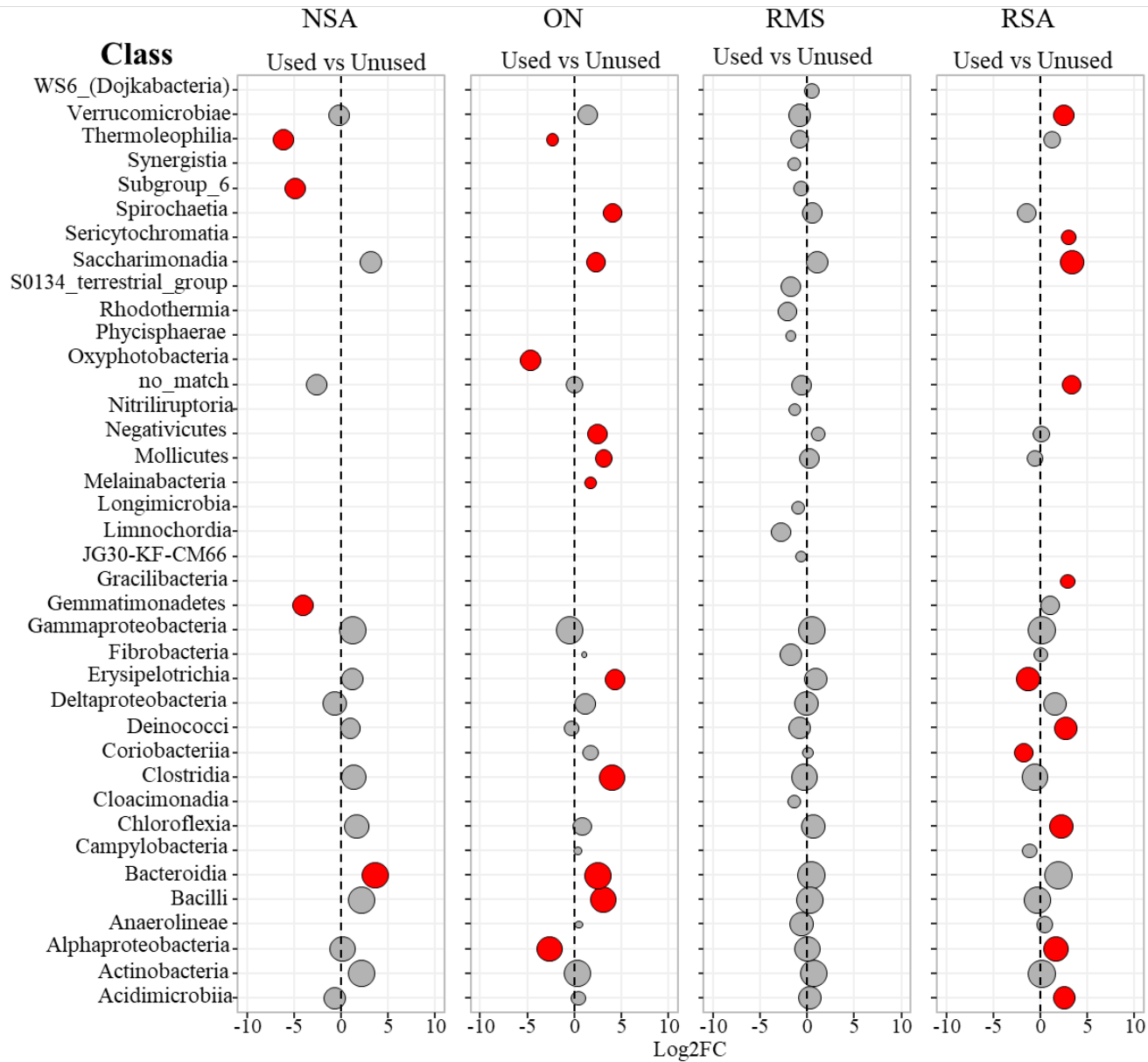

Figure S3

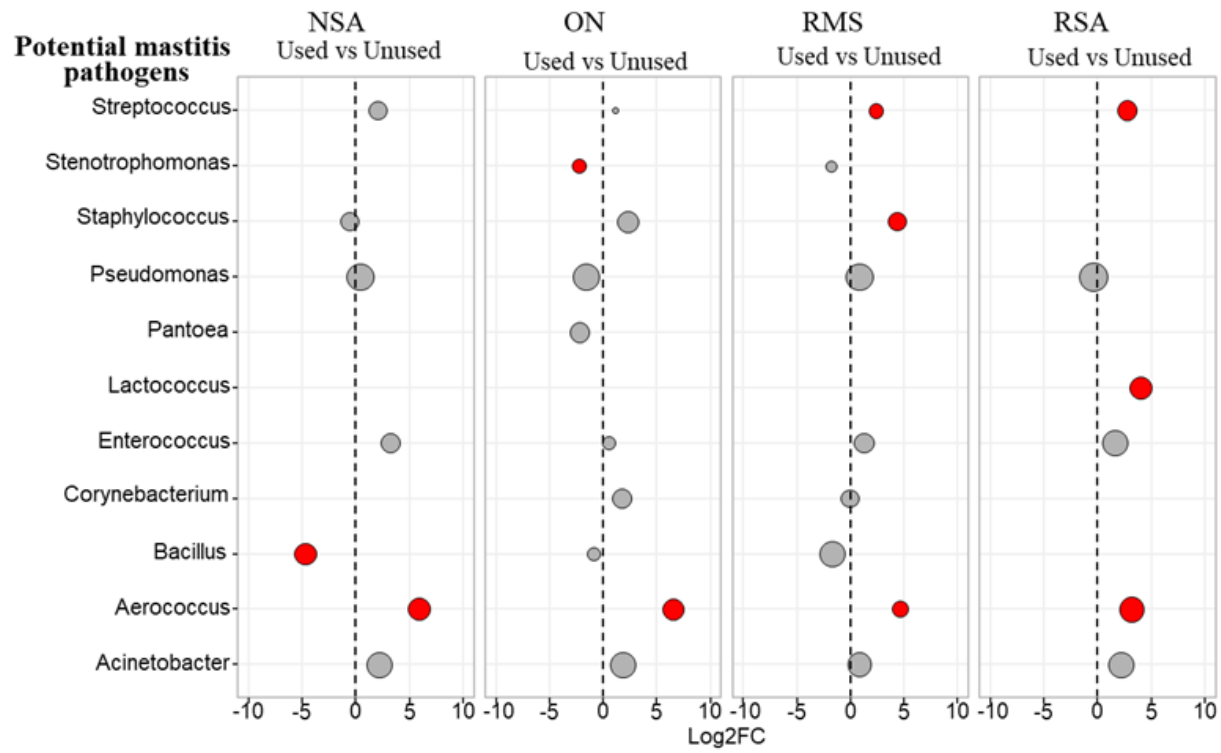

Figure S4

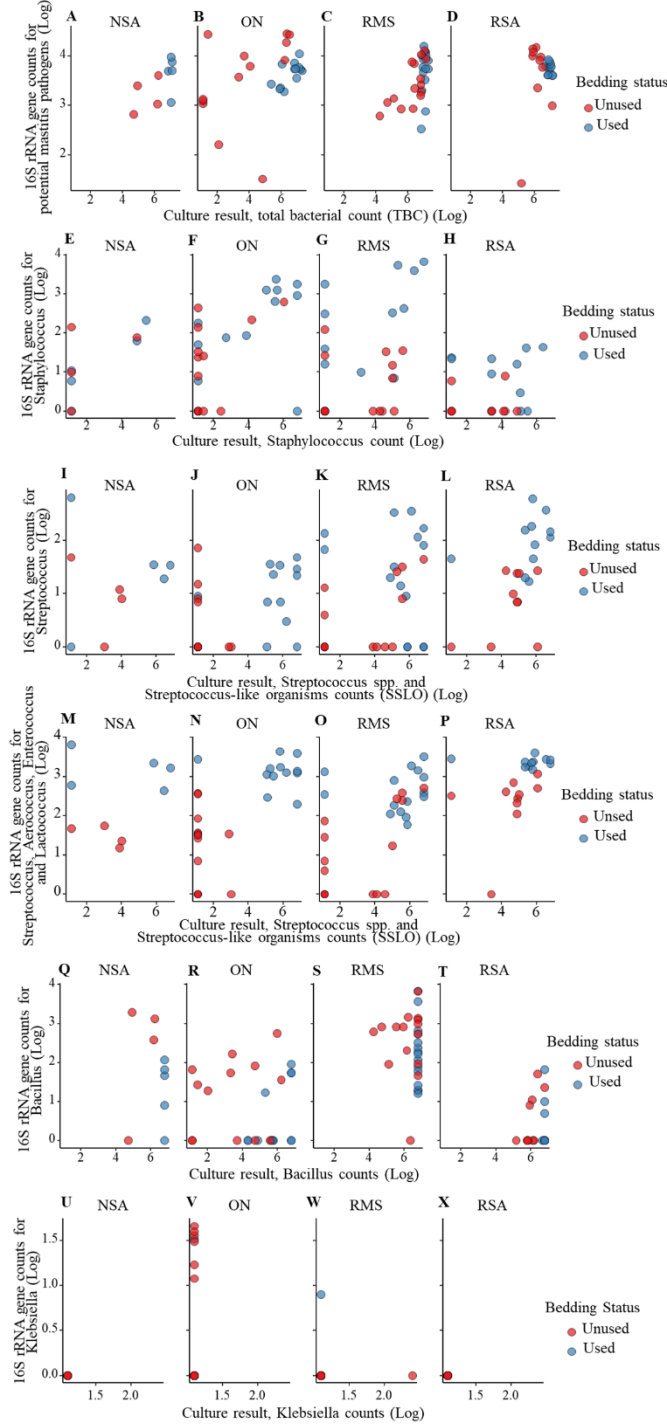

Supplement: Supplementary file 2 — Additional file 2: Fig. S1. Non-metric multidimensional scaling (NMDS) ordination plots based on Bray–Curtis distances for (A) used versus (B) unused status for each bedding type, at the phylum, class and ASV level; and for potential mastitis pathogens at the genus level. NSA—new sand, ON—organic non-manure, RMS—recycled manure solids and RSA—recycled sand bedding type. Fig. S2. Log2-fold change (Log2FC) in abundance of classes between used and unused bedding samples, separated by bedding type. Only classes with an average abundance >50th percentile within each bedding type are depicted. Red indicates classes whose abundance was significantly different between used and unused bedding samples (i.e., adjusted P < 0.05). Circle diameter is proportional to the average abundance of each genus across all samples within each bedding type. NSA—new sand, ON—organic non-manure, RMS—recycled manure solids and RSA—recycled sand bedding type. Fig. S3. Log2-fold change (Log2FC) in abundance of genera that contain potential mastitis pathogens, comparing used and unused bedding samples, separated by bedding type. Only genera with an average abundance >50th percentile within each bedding type are depicted. Red indicates genera whose abundance was significantly different between used and unused bedding samples (i.e., adjusted P < 0.05). Circle diameter is proportional to the average abundance of each genus across all samples within each bedding type. NSA—new sand, ON—organic non-manure, RMS—recycled manure solids and RSA—recycled sand bedding type. Fig. S4. Scatter plots of 16S rRNA gene counts and culture results obtained from the same bedding samples, for: total bacteria (panels A–D, TBC); Staphylococcus (panels E–H); 16S rRNA gene counts for Streptococcus and culture-based Streptococcus and Streptococcus like organisms (SSLO) counts (panels I–L); 16S rRNA gene counts for Streptococcus, Aerococcus, Enterococcus and Lactococcus and culture-based Streptococcus and Streptococcus like [file 42523_2022_171_MOESM2_ESM.pdf]
